# Intracranial hemorrhage due to central venous occlusion from hemodialysis access: A case report

**DOI:** 10.1016/j.inat.2020.101081

**Published:** 2021-01-04

**Authors:** Mohammed H. Mirza, Adam Schwertner, Ryan Kohlbrenner, Christopher F. Dowd, Kazim H. Narsinh

**Affiliations:** a Department of Radiology, University of Illinois College of Medicine, Peoria, IL, United States; b Department of Radiology, University of Colorado Denver School of Medicine, Denver, CO, United States; c Department of Radiology & Biomedical Imaging, University of California San Francisco, San Francisco, CA, United States

**Keywords:** Intracranial hemorrhage, Intracranial venous hypertension, Central venous occlusion

## Abstract

Central venous stenosis in hemodialysis patients rarely causes venous hypertension and intracranial hemorrhage. A 54 year-old male with right arm arteriovenous fistula was transferred to our institution in a comatose state following right parietal venous infarction. Fistulography showed right brachiocephalic vein (BCV) occlusion with reflux into the right transverse sinus and obstruction of left internal jugular vein outflow due to the styloid process. Balloon venoplasty of the right BCV occlusion failed to improve the patient’s status because of the delayed diagnosis. Headaches and neurologic symptoms in hemodialysis patients can herald intracranial hypertension due to central venous occlusion and needs prompt assessment with fistulography.

## 1. Introduction

End-stage renal disease (ESRD) patients develop central venous stenosis (CVS) 25–40% of the time due to prolonged need for hemodialysis (HD) access using arm arteriovenous fistulas (AVFs) or central venous catheters (CVCs) [1]. While most central venous stenoses remain asymptomatic [2], patients can develop facial, neck, or arm pain and edema that prompt them to seek medical attention. Less commonly, central venous stenosis can lead to intracranial venous hypertension, causing symptoms such as headache, cognitive impairment, or tinnitus. Because of the difficulty making this diagnosis due to the nonspecific neurologic complaints, intracranial venous hypertension has a morbid clinical course. If left untreated, intracranial hypertension can result in venous cerebral infarction [3]. Herein, we report a case of an ESRD patient on HD who developed intracranial hypertension due to central venous occlusion, highlighting the presentation, natural history, and treatment of this clinical condition. Prompt recognition and endovascular treatment can prevent intracranial hemorrhage.

## 2. Case history

**Fig. 1.**
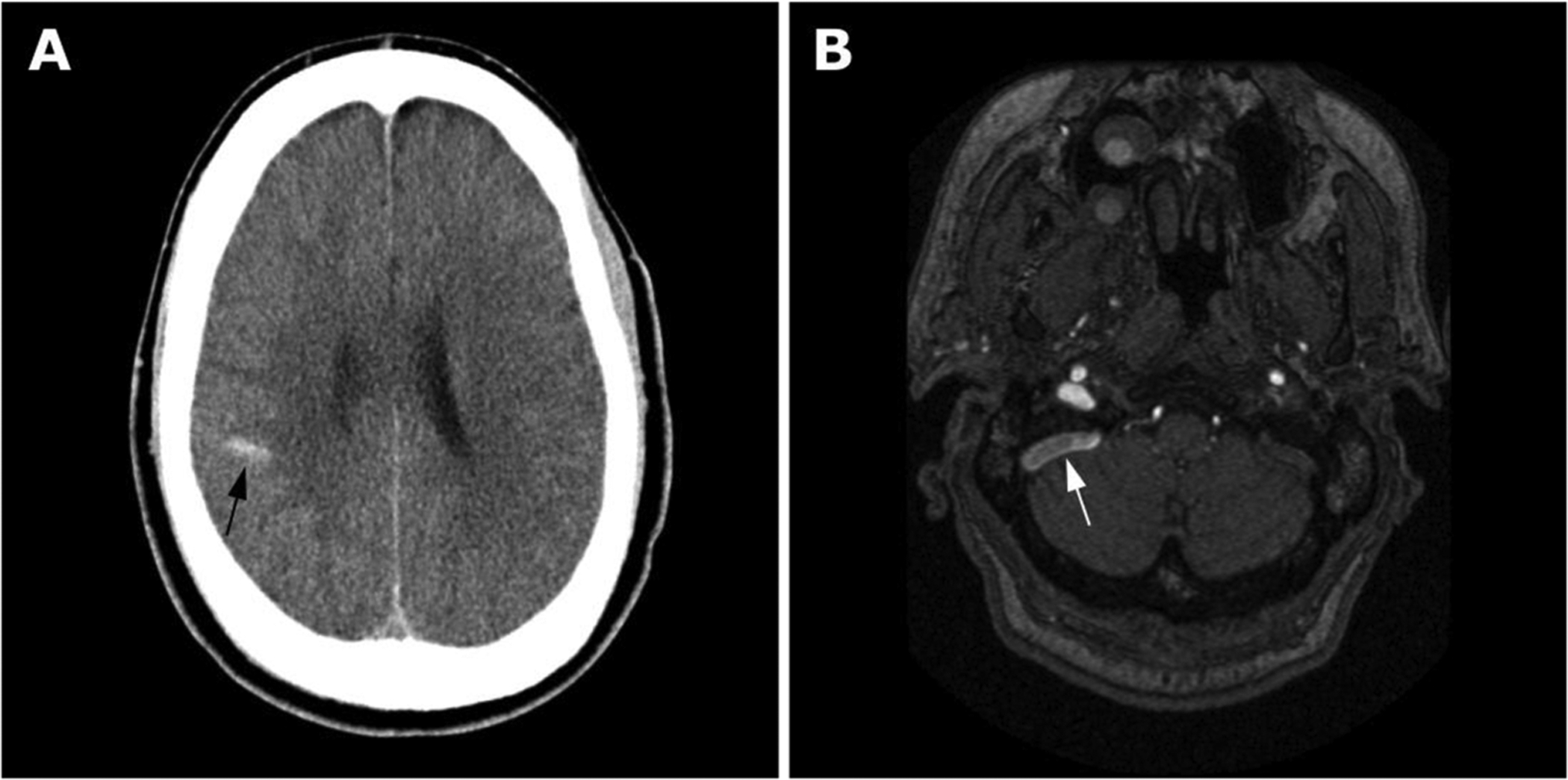
(A) Axial head CT reveals right posterior temporal-parietal hemorrhage (black arrow). (B) Axial time-of-flight MRA demonstrates flow-related enhancement in the right sigmoid sinus (white arrow) consistent with venous reflux in cranial direction.

A 54-year-old male with ESRD on HD via a right upper arm AVF was admitted to an outside hospital with right internal jugular vein thrombosis, right parietal venous infarct, subarachnoid hemorrhage, and seizure (Fig. 1A). Prior to presentation, the patient was not taking any antithrombotic medications, and anticoagulation was not administered to treat the jugular vein thrombus on account of religious objection to blood transfusion and elevated bleeding risk due to anemia of chronic disease. Limited hypercoagulability labs revealed elevated homocysteine but were otherwise unremarkable. Lumbar puncture (LP) indicated elevated opening pressure of 55 cm H_2_O that improved significantly on repeat LP. CSF diversion was not pursued because an underlying cause of intracranial hypertension was suspected. He was subsequently discharged to a skilled nursing facility with a plan for outpatient management of intracranial hypertension, including repeat LP. At the skilled nursing facility, he had intermittent non-positional headaches but no facial or arm pain.

Due to his headaches and previous intracranial hemorrhage, brain MRI/MRA/MRV was obtained, demonstrating flow-related enhancement in the right sigmoid sinus (Fig. 1B) and sequelae of prior right parietal venous infarction. Catheter arteriography and venography with manometry were recommended out of concern for intracranial dural arteriovenous fistula (dAVF), but the patient was unable to come to the clinic appointment required prior to the procedure.

**Fig. 2.**
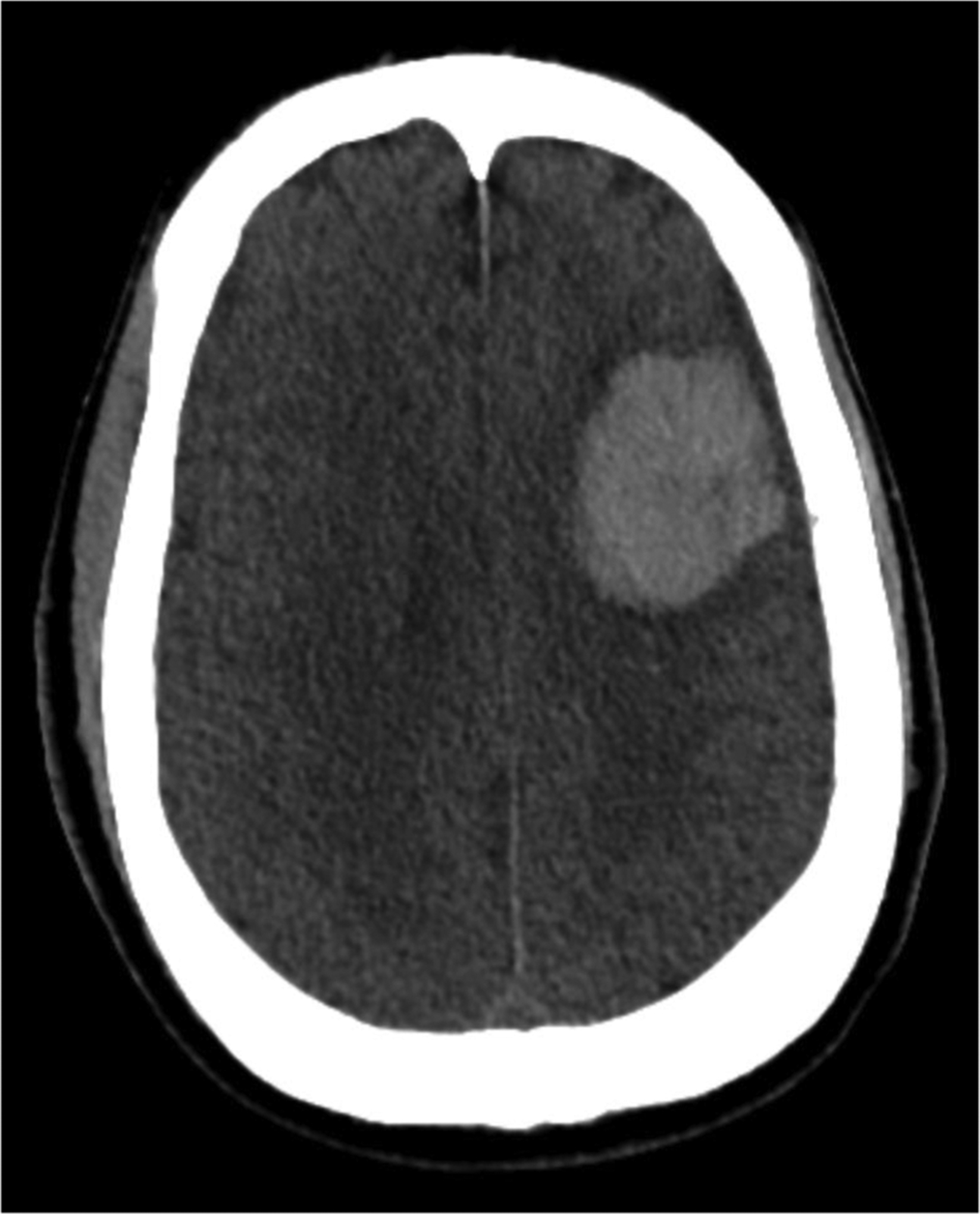
Axial head CT shows a 4 cm left frontal lobe intraparenchymal hemorrhage.

One month later, the patient experienced left arm weakness and confusion. He presented with encephalopathy and diminished arousability during hemodialysis that progressed to a comatose state. Neurologically, he maintained brainstem reflexes and withdrawal to noxious stimuli, and he had no noticeable erythema, venous engorgement, or edema in his face, neck, and arm. The patient was intubated and had multiple seizures during admission. Brain MRI showed multiple foci of microhemorrhage and vasogenic edema. Lumbar puncture had opening and closing pressures greater than 50 cm H_2_O despite 18 mL CSF drainage. Head CT (Fig. 2) showed a new 4 cm left frontal lobe intraparenchymal hemorrhage (IPH), and he was transferred to our institution for further evaluation and management of suspected dAVF.

## 3. Investigation (imaging studies)

**Fig. 3.**
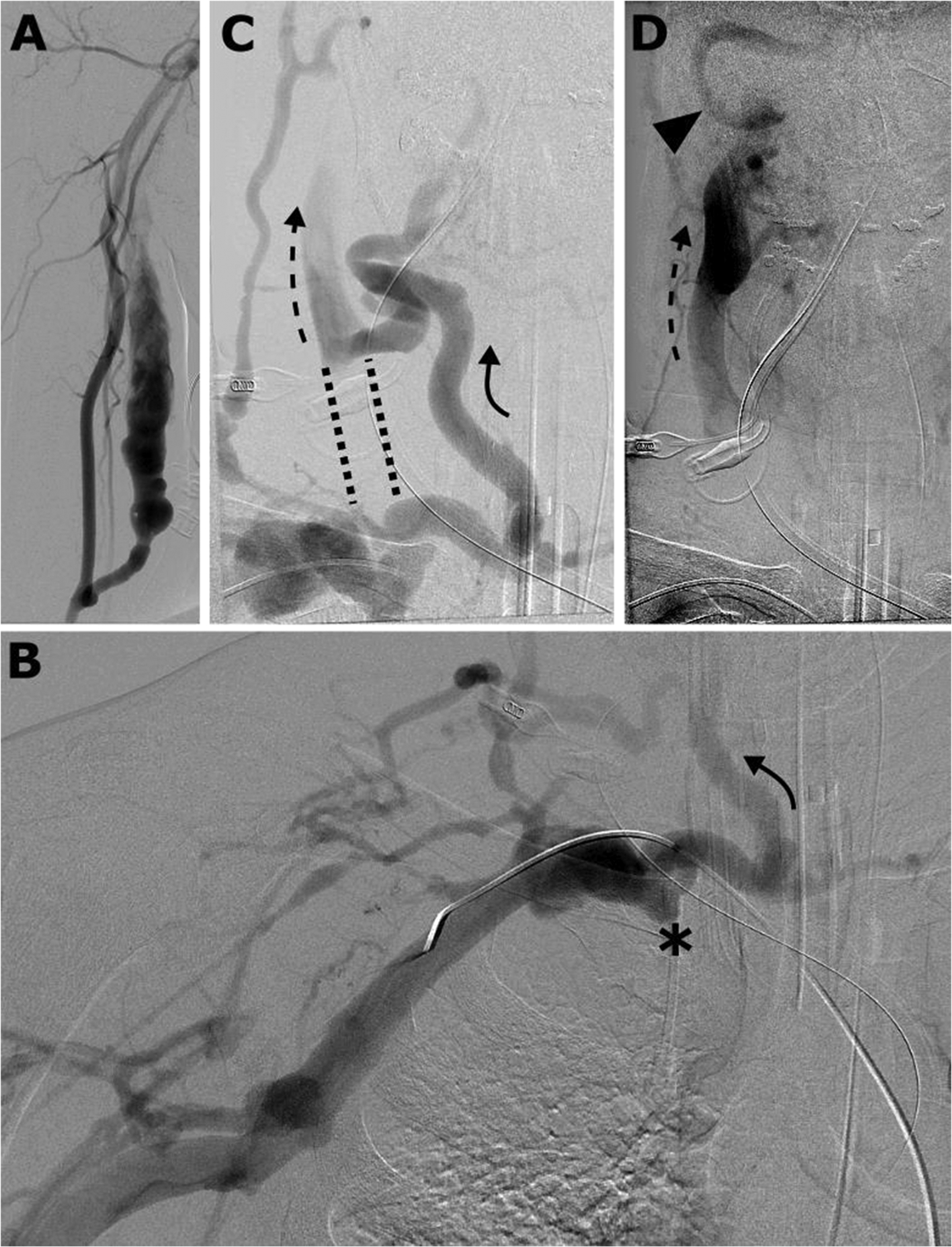
(A) Transarterial fistulogram demonstrates a patent right upper arm brachial to transposed basilic AVF without perianastomotic or venous outflow stenosis. (B) Right brachiocephalic vein occlusion (asterisk) is seen in the venous phase, with collateral flow into a right anterior jugular vein (curved arrow). (C) Venous reflux into the right anterior jugular vein (curved arrow) continues cranially into the right internal jugular vein (dashed arrow). The central portion of the right internal jugular vein is occluded (dashed lines). (D) Reflux into the internal jugular vein (dashed arrow) continues cranially into the right sigmoid sinus (arrowhead).

Upon transfer, the patient presented with brainstem reflexes and asymmetric grimace. Non-contrast head CT (NCHCT) revealed a 3.8 × 2.3 × 3.9 cm left frontal IPH (Fig. 2), with a positive “spot sign” on CTA and intraventricular extension, as well as petechial hemorrhage in right superior temporal lobe and right inferior frontal lobe. Evacuation of the hematoma was not performed because of lack of midline shift or transtentorial herniation. Subsequent CTA revealed an acute right internal jugular vein (IJV) occlusion and chronic occlusion of right brachiocephalic vein. Further evaluation focused on the cause of his intracranial hypertension. Cerebral angiogram showed no dural AVF. Transarterial fistulogram was then performed to evaluate for other potential causes of intracranial hypertension. He had a patent right upper arm brachial to transposed basilic AVF without perianastomotic or venous outflow stenosis (Fig. 3A). Venous phase of the transarterial fistulogram demonstrated central venous occlusion of the right brachiocephalic vein and central right IJV, with reflux of the AVF outflow cephalad via a right anterior jugular vein collateral into the right internal jugular vein and then intracranially into the right sigmoid and transverse sinuses (Fig. 3B–D) as well as partial obstruction of the left IJV across the styloid process.

Transcatheter venous pressure measurements demonstrated elevated left sigmoid sinus venous pressure of 24 mm Hg (reduced to 17 mm Hg with compression of RUE AVF), compatible with intracranial venous hypertension. Stenosis of the high left internal jugular vein due to the left styloid process was confirmed by a 17 mm Hg pressure gradient and reduction of the pressure gradient by 7 mm Hg with rightward head turn and neck extension.

## 4. Treatment

**Fig. 4.**
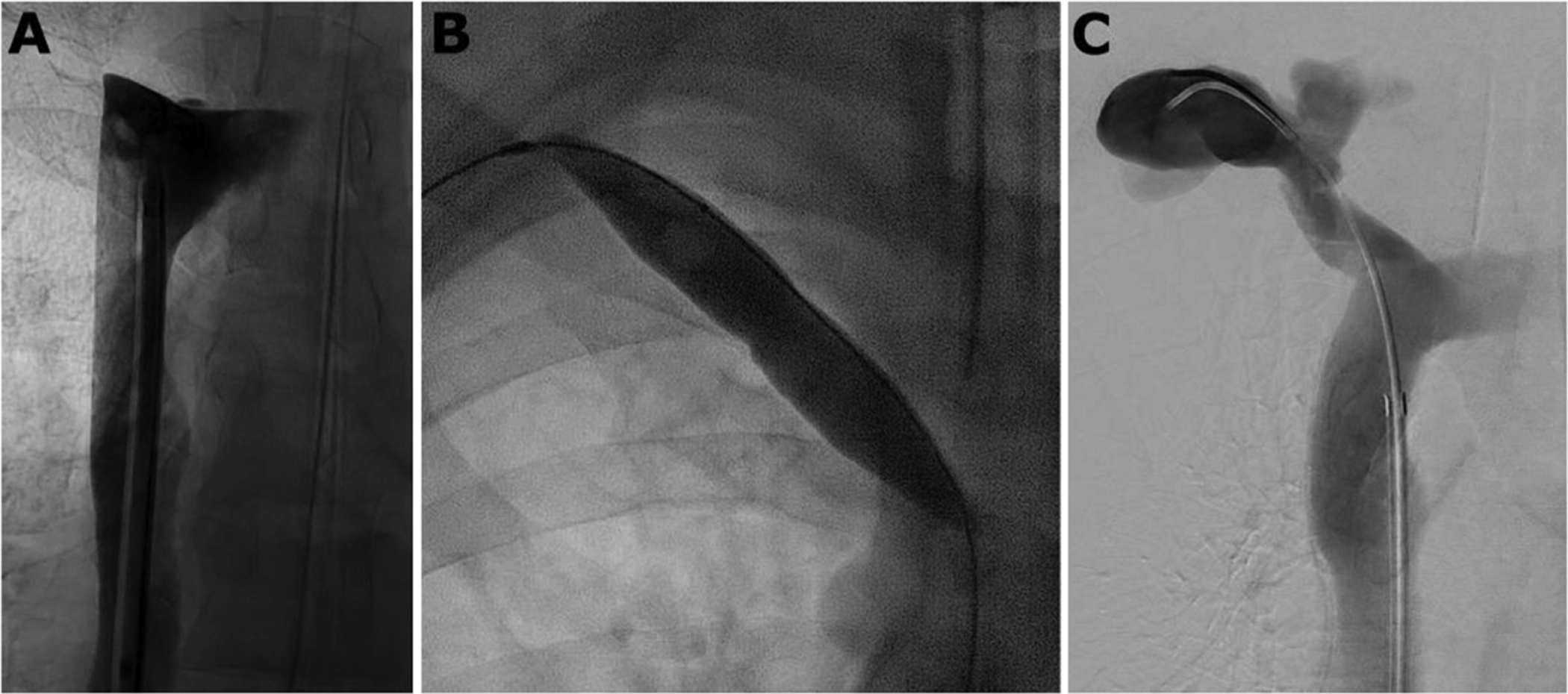
(A) Superior vena cavogram demonstrating central aspect of the occluded right brachiocephalic vein. (B) Venoplasty to 16 mm using a high-pressure noncompliant balloon. (C) Post-venoplasty venography demonstrated excellent luminal gain in the right brachiocephalic vein with resolution of collateral flow.

Transfemoral venography demonstrated the lower aspect of the occluded right brachiocephalic vein. After recanalizing the occlusion with a hydrophilic guidewire and 4 Fr catheter, serial venoplasty was performed using 4 mm–16 mm high-pressure noncompliant balloons. After venoplasty, venography demonstrated excellent luminal gain and central venous drainage of the right arm AVF outflow (Fig. 4).

## 5. Outcome

Seven days after venous recanalization, the patient’s neurologic examination deteriorated. An external ventricular drain was placed, followed by an intracranial pressure monitor due to unreliable ICP waveforms. The patient’s intracranial pressure remained labile post procedure – spiking to the 40–50s cm H_2_O range – prompting hyperosmolar therapy. Despite increased hyperosmolar and multiple pressor therapy, the patient developed refractory hemodynamic instability. Given his poor neurologic exam and grave prognosis, craniotomy and hematoma evacuation were not recommended, and the patient died ten days after venous recanalization.

## 6. Discussion

While CVS is a significant problem associated with dialysis access, its precise incidence still remains relatively obscure on account of limited imaging of the general ESRD patient population [4,5]. Rarely, CVS results in intracranial venous hypertension, which has a morbid clinical course. Symptomatic patients are especially notable for presenting with complaints of focal pain and peripheral concerns of grossly appreciable edema and venous engorgement. Yet, in the absence of such clinical signs and symptoms, development of nonspecific neurologic complaints can herald the development of intracranial hypertension with potentially lethal consequences, thus posing a grave diagnostic challenge.

Albeit multifactorial, the pathogenesis of CVS is postulated to arise principally due to central venous catheter-induced intraluminal micro-trauma and turbulent intravenous blood flow due to arteriovenous shunting causing intimal hyperplasia [6,7]. In the event of a severe obstruction, central venous occlusion (CVO) can cause internal jugular venous reflux, which can typically be compensated by drainage across the midline torcular herophili into the contralateral jugular vein and back to the right atrium. However, when the contralateral jugular vein is also obstructed, such as by the styloid process, intracranial hypertension can develop, and such cerebral venous hypertension puts the hemodialysis patient at significant risk of suffering intracranial hemorrhage, venous infarction, and seizures [3,6,8,9].

In this case, obstruction of the contralateral jugular vein was confirmed by measurement of a pressure gradient across the styloid process. Obstruction of left internal jugular vein outflow across the styloid process has sometimes been referred to as Eagle syndrome. Also known as stylohyoid syndrome, Eagle syndrome is associated with an elongated styloid process capable of stretching nerve endings and/or impinging on the carotid artery leading to pain and cerebral ischemia, respectively. It has also been shown in the literature to cause significant compression of the internal jugular vein with reported sequelae similar to those described above: venous congestion, intracranial hypertension, and cerebral hemorrhage [10]. Thus, in the context of obstruction of both jugular veins, this patient likely experienced severe intracranial venous congestion and thereafter developed multicompartmental bilateral intracranial hemorrhage.

Much emphasis has been put on the prevention of symptomatic CVS/CVO in HD patients, including the preference of fistulas over grafts and catheters, forearm access over upper arm access, and right-sided access over left-sided access [4,11]. The diagnosis of CVS/CVO can be established with venography, and lumbar puncture with an opening pressure can confirm intracranial hypertension [6]. Measuring venous pressures during venography can also quantitatively assess venous hypertension, and post-treatment measurements can be used to confirm the success of intervention [12].

Treatment of intracranial hypertension symptoms includes medical management with intracranial pressure-lowering agents (e.g. acetazolamide, mannitol) and CSF drainage. However, definitive treatment is aimed at treatment of the underlying cause. Both dAVF and CVS/CVO can have similar noninvasive imaging findings, and these need to be distinguished on catheter angiography. Treatment success depends heavily upon early detection. Prompt discovery and intervention – especially before the onset of hemorrhage – has been shown to effectively allow for the resolution of focal neurological deficits [3,8,9,12]. Nevertheless, timely diagnosis of venous congestive encephalopathy proves especially difficult considering its non-specific – and sometimes neurologically unremarkable – presentations such as intermittent headaches early in the disease course [8].

ESRD patients on HD who have new-onset headaches or neurological symptoms merit prompt attention for assessment of intracranial hypertension induced by CVO/CVS. Intracranial venous hypertension has a morbid clinical course due to the high rate of progression to intracranial hemorrhage.
